# Association of Copper Status with Lipid Profile and Functional Status in Patients with Amyotrophic Lateral Sclerosis

**DOI:** 10.1155/2018/5678698

**Published:** 2018-07-19

**Authors:** Acsa Nara A. B. Barros, Mário Emílio T. Dourado, Lucia de Fatima C. Pedrosa, Lucia Leite-Lais

**Affiliations:** ^1^Department of Nutrition, Federal University of Rio Grande do Norte, Natal, RN, Brazil; ^2^Department of Medicine, Federal University of Rio Grande do Norte, Natal, RN, Brazil

## Abstract

Oxidative stress is one of the main mechanisms associated with the pathogenesis of amyotrophic lateral sclerosis (ALS). Copper can affect cellular oxidation and lipid metabolism. The aim of this study was to evaluate the association of copper status with lipid profile and functional status in patients with ALS. A cross-sectional study was carried out including 27 patients with ALS (case group) and 26 healthy individuals (control group). Copper status was evaluated by habitual dietary copper intake, plasma copper, and serum ceruloplasmin concentrations. The lipid profile included analysis of serum total cholesterol (TC), LDL-cholesterol (LDL-c), HDL-cholesterol (HDL-c), and triglycerides (TGL). The functional status of patients with ALS was assessed by the ALS Functional Rating Scale-Revised (ALSFRS-R). In the case group, plasma copper was lower compared with the control group (133.9 versus 164.1 *μ*g/dL, *p*=0.0001) and was positively correlated with HDL-c (*r*_s_=0.398, *p*=0.044). In the control group, plasma copper was positively correlated with serum ceruloplasmin (*r*_s_=0.646, *p* < 0.001), TC (*r*_s_=0.446, *p*=0.025), LDL-c (*r*_s_=0.445, *p*=0.029), and HDL-c (*r*_s_=0.479, *p*=0.015), and serum ceruloplasmin was positively correlated only with LDL-c (*r*_s_=0.407, *p*=0.043). In the case group, dietary copper intake (*B*=−0.373, *p* < 0.001), plasma copper (*B*=−0.005, *p*=0.033), and TC (*B*=−0.312, *p*=0.001) were inversely associated with the functional status of patients with ALS. In contrast, serum ceruloplasmin (*B*=0.016, *p*=0.044), LDL-c (*B*=0.314, *p*=0.001), HDL-c (*B*=0.308, *p*=0.001), and TGL (*B*=0.062; *p*=0.001) were positively associated with their functional status. In conclusion, this study suggests a disturbance of copper status and its connection with the lipid profile in patients with ALS. Furthermore, copper status and lipid profile may influence the functional status of patients with ALS, standing out as potential biomarkers of disease severity.

## 1. Introduction

Amyotrophic lateral sclerosis (ALS) is a progressive and fatal disease characterized by degeneration of motor neurons in the brain and spinal cord, leading to skeletal muscle atrophy, paralysis, and death. The incidence of ALS is about 1/100,000, with a survival time since onset ranging from 24 to 48 months and an estimated mortality of 30,000 patients a year worldwide [1]. The etiology of ALS remains unknown. However, several mechanisms have been implicated in ALS pathophysiology and progression, such as oxidative stress, glutamate excitotoxicity, mitochondrial dysfunction, neuroinflammation, and protein aggregation [2].

Abnormal metal homeostasis may have an important role in the onset and progression of neurodegenerative disorders [3]. In this sense, copper has been extensively studied and plays an important role in the central nervous system (CNS). This micronutrient is essential for angiogenesis, myelination, neurotransmission, cellular respiration, and antioxidant defense. Thus, disturbances in copper homeostasis generate functional impairments in the CNS, including neurodegeneration [4], one of the characteristics found in ALS. In addition, both copper excess and deficiency contribute to oxidative stress, a key component of ALS progression [5]. Copper excess catalyzes biochemical reactions that produce reactive oxygen species. Copper deficiency, in turn, leads to poor activity of copper-dependent antioxidant enzymes, such as superoxide dismutase (SOD), glutathione peroxidase (GSH-Px), and metallothionein [6]. Copper-mediated oxidative stress is associated with changes in genes expression, lipoprotein structure, membrane receptors, and consequently lipid profile [7].

Some studies have demonstrated that higher serum lipid concentrations are associated with slower progression, better prognosis, and prolonged survival in ALS patients [8–11]. The link between copper status and lipid metabolism has been reviewed [7, 12, 13]. Clinical studies have found different associations between serum copper and lipids, depending on the population studied and the presence of comorbidities [14–19]. Thus, both copper excess and deficiency not only contribute to oxidative stress but also alter serum lipid concentrations [7].

Copper status and lipid profile have been studied in ALS patients separately; however, these two subjects have not been investigated together in these patients. Considering the scarcity of studies on this subject, the present study aimed at evaluating the association of copper status with lipid profile and functional status in patients with ALS.

## 2. Materials and Methods

### 2.1. Participants and Study Design

This cross-sectional study was reviewed and approved by the Ethics Committee of the Onofre Lopes University Hospital in Natal, Brazil (CAAE 40467214.0.0000.5292). All subjects provided written, informed consent before enrollment.

The case group consisted of patients with ALS treated at the multidisciplinary outpatient facility at the Onofre Lopes University Hospital in Natal, Brazil, between March 2016 and December 2016. Inclusion criteria were patients of both sexes with probable or definite ALS diagnosis and under the care of a literate family member or caregiver. Exclusion criteria were patients with suspected or possible ALS diagnosis, taking micronutrient supplements, undergoing estrogen treatment, and with another neurological disease, inflammatory bowel disease, biliary diseases, thyroid dysfunction, diabetes mellitus, and renal and hepatic diseases. These exclusion criteria were adopted due to their possible influence on copper status.

For comparison purposes, healthy adults and elderly individuals were recruited as the control group. The recruitment of the control group occurred through social media invitations sent to the local community. A matching based on age and sex was adopted to improve the quality of the data. The same exclusion criteria were adopted for the control group.

### 2.2. Clinical Characterization

Patients with ALS were characterized by the onset site of symptoms (bulbar or spinal), time of symptoms (in months), feeding route (oral and/or enteral), and functional status using the ALS Functional Rating Scale-Revised (ALSFRS-R) [20] and validated to the Portuguese language [21]. This instrument evaluates the progression of patients' disability and the severity of the disease through a 12-item questionnaire, covering aspects related to dysphagia, daily life activities, and respiratory function. The overall ALSFRS-R score ranged from 0 to 48, where 0 represents the worst stage of disability and 48 represents the normal functional status.

### 2.3. Anthropometric Assessment

The anthropometric assessment was performed utilizing the body mass index (BMI) [22]. The body weight was measured in a calibrated digital Knwaagen® scale with maximum capacity of 500 kg. The height was measured using a Medjet® stadiometer. For wheelchair patients, the height was estimated according to Chumlea et al. [23, 24].

### 2.4. Copper Status and Lipid Profile Evaluation

#### 2.4.1. Dietary Copper Intake

Habitual dietary copper intake was investigated using two nonconsecutive 24-hour recalls for each participant, obtained from weekdays, 30 to 45 days apart [25]. Food intake data were calculated using Virtual Nutri Plus® 2.0 software. Food items or preparations that were not found in the software's databank were added based on the nutrition labels of industrialized products. Habitual dietary copper intake was estimated after adjustments of interpersonal variability, according to Nusser et al. [26]. Next, copper intake was adjusted for energy, applying the residual method described by Willett et al. [27]. The estimated average requirement (EAR) for copper (0.7 mg/day) [28] was used as a parameter to assess copper intake.

#### 2.4.2. Plasma Copper, Serum Ceruloplasmin, and Lipid Profile

After an overnight fast, blood samples were collected from participants in specific tubes to perform all hematological analysis. For plasma copper evaluation, blood samples were collected in metal-free tubes with 100 *μ*L of 30% sodium citrate anticoagulant. For serum ceruloplasmin evaluation, blood samples were collected in tubes with gel clot activator (BD Vacutainer® SST™ II Advance, BD, UK). For lipid profile evaluation, blood samples were collected in tubes with gel clot activator (VacuTube, Biocon, BR).

Plasma copper concentrations were determined by atomic absorption spectrophotometry (SpectrAA200, Varian, Victoria, Australia). The samples were centrifuged at 3000 rpm for 15 minutes at 4°C, and the plasma was separated and stored in a freezer at −20°C until analysis. The calibration curves were prepared with Titrisol® standard solution (Merck, Germany), at the following concentrations: 0.00, 0.10, 0.20, 0.30, 0.50, and 1.00 *μ*g/mL. Aliquots of 600 *μ*L of plasma were diluted in deionized water at 1 : 5 ratio. After reading, the results were expressed as *μ*g/dL. Analysis was conducted in duplicate, and the results were calculated from the average of the readings, establishing a coefficient of variation of less than 10%. A range of 70–140 *μ*g/dL was considered as the reference value for plasma copper for both sexes [29].

Analysis of serum ceruloplasmin and lipid profile was performed in a private certified laboratory. The nephelometry method was used to measure serum ceruloplasmin and 21–53 mg/dL was considered as the reference range for both sexes. Serum total cholesterol (TC) and triglycerides (TGL) were measured by the enzymatic method and HDL-cholesterol (HDL-c) by direct calorimetric method. The LDL-cholesterol values (LDL-c) were calculated using the Friedewald formula [30].

### 2.5. Statistical Analysis

The descriptive analysis was performed using measures of central tendency and dispersion, according to the data type. The Shapiro–Wilk test was applied to verify the normality of the data. To verify differences between the continuous variables, Student's *t*-test and Wilcoxon–Mann–Whitney test were used. The inferential analysis was performed by estimating the correlation between copper status and the lipid profile by Spearman correlation test.

The effect of independent variables on the outcome (discrete), measured by the ALSFRS-R score, was estimated after the univariate analysis. An adjusted model based on the Poisson distribution was used, as recommended for discrete outcomes in transversal studies. Dietary copper, plasma copper, serum ceruloplasmin, TC, LDL-c, HDL-c, and TGL were considered independent variables.

The cutoff point for the inclusion of the variables in the adjusted final model was *p* < 0.30. In order to accept the final model, its significance was assessed using the chi-square test phrased as likelihood ratio. The variables were assessed separately by Wald chi-square test to estimate the regression coefficients. The presence of normality was tested for residues to guarantee the validity of the model. Statistical analysis was performed using SPSS v.22, and a significance level of 5% was adopted for all analyses.

## 3. Results

Of the 53 subjects enrolled in the study, 27 comprised the case group and 26 the control group. The mean age of the participants in the case and control groups was 56 (12.6) and 55.4 (12.5) years, respectively. The case group presented a significantly lower BMI (*p* < 0.0001) compared with the control group. The case group had a disease time of 46 (22–72) months, and 60% of the patients had ≤24 points in the ALSFRS-R score (Table 1).

Dietary copper intake did not differ significantly between the groups (*p*=0.4122). Also, the values of copper biomarkers and lipid profile were similar between the groups. However, plasma copper concentrations in the case group were significantly lower than those in the control group (133.9 versus 164.1 *μ*g/dL, *p*=0.0001) (Table 1).

In the case group, plasma copper was positively correlated only with HDL-c (*r*_s_=0.398, *p*=0.044). In the control group, plasma copper was positively correlated not only with HDL-c (*r*_s_=0.479, *p*=0.015) but also with serum ceruloplasmin (*r*_s_=0.646, *p* < 0.001), TC (*r*_s_=0.446, *p*=0.025), and LDL-c (*r*_s_=0.445, *p*=0.029). Serum ceruloplasmin was positively correlated only with the LDL-c in the control group (*r*_s_=0.407, *p*=0.043). Neither plasma copper nor serum ceruloplasmin was correlated with dietary copper (Table 2).

In the case group, dietary copper (*B*=−0.373, *p* < 0.001), plasma copper (*B*=−0.005, *p*=0.033), and TC (*B*=−0.312, *p*=0.001) were inversely associated with the ALSFRS-R score. Among them, dietary copper had the strongest association. On the other hand, serum ceruloplasmin (*B*=0.016, *p*=0.044), LDL-c (*B*=0.314, *p*=0.001), HDL-c (*B*=0.308, *p*=0.001), and TGL (*B*=0.062, *p*=0.001) were positively associated with the ALSFRS-R score (Table 3).

## 4. Discussion

The case group presented a significantly lower mean BMI compared with the control group. Weight loss and reduced BMI are associated with accelerated disease progression and lower survival rate [31]. Spinal-onset ALS, as found in most patients of this study, has less impairment in the swallowing ability than bulbar-onset ALS. This explains the use of enteral nutrition by the minority of the patients studied. The majority of patients presented low ALSFRS-R score, representing high disability and disease severity.

Our results showed that there was no difference in dietary copper intake between the groups. In addition, the median of dietary copper intake in the case and control groups was in agreement with the recommendations adopted [28]. Park et al. [32] found that the dietary copper intake among patients with ALS ranged from 0.9 mg/day to 1.2 mg/day. In our study, this variation was broader (Table 1), demonstrating a greater copper supply by the diet.

Contrary to our results, Forte et al. [33] found a significantly higher concentration of blood copper in patients with ALS (103.5 *μ*g/dL) compared with the control group (95.4 *μ*g/dL). However, other studies found no significant difference in plasma copper concentrations between ALS patients and healthy subjects [34, 35]. Differences in blood copper concentrations between studies may be explained by differences in food eating pattern, molecular profile of the population studied, and the method used to perform the lab tests. Considering the clinical heterogeneity among ALS patients, Forte et al. [33] suggest that the reference range for blood copper concentrations in subjects with and without ALS should be reviewed.

Mineral metabolism disturbances in patients with ALS may be due to several factors, such as malnutrition, malabsorption, increased excretion, and competition between minerals at binding sites [34]. Also, oxidative stress in patients with ALS may alter copper homeostasis [6, 36] and contribute to neurodegeneration [37]. At the same time, copper-deficient SOD-1 has a loss of antioxidant function and exerts a prooxidant function [38]. In addition, the level of copper deficiency seems to be proportional to the clinical severity of ALS [39].

In agreement with our results, some authors did not observe significant differences in the lipid profile between patients with ALS and healthy individuals [8, 40]. Interestingly, some authors found significantly lower concentrations of TGL in male, but not in female, individuals with ALS compared with their control group [41]. Although there was no difference of serum lipids between males and females in both groups of our study (data not shown), Ikeda et al. [42] found significantly higher concentrations of TC, LDL-c, and TGL in female Japanese patients with ALS, compared with male ones. These controversial results may be explained by several genetic polymorphisms associated with lipid metabolism, able to develop individual biochemical phenotypes [43].

No correlation of dietary copper with plasma copper and serum ceruloplasmin was observed in both groups studied. In a recent review, Bost et al. [44] found that biomarkers of copper not necessarily respond to changes in dietary copper intake, probably due to homeostatic mechanisms controlling the absorption and excretion of this mineral. Also, there was no correlation between plasma copper and serum ceruloplasmin in the case group, but this correlation was positive in the control group. In healthy subjects, we expect to find a positive correlation between copper and ceruloplasmin, which is the main copper-binding protein [45]. Patients with ALS may have disturbances of ceruloplasmin activity [46] and lack of copper incorporation during ceruloplasmin biosynthesis [47], weakening a possible correlation between these two parameters.

In our study, the only correlation found between the copper biomarkers and the lipid profile in the case group was a positive correlation between plasma copper and HDL-c. It is possible that changes in serum lipids, oxidative stress, and alterations in copper metabolism in ALS are responsible for the absence of correlation between copper and serum lipids, differing from what was found in the control group. Several studies have found different associations between serum or plasma copper and serum lipids, demonstrating great metabolomic variation among individuals [14–19]. Circulating lipids are influenced by genetics, metabolism, habitual diet, lifestyle, and their interactions [48]. Moreover, disturbance of copper metabolism influences serum lipids through oxidative stress [49–51].

In our study, the association of ALSFRS-R with copper and lipid biomarkers was very divergent (Table 3). Dietary copper and plasma copper were inversely associated, while serum ceruloplasmin was positively associated with the functional status of ALS patients. Authors have found positive association between the concentration of copper in the nails of patients with ALS and functional status assessed by ALSFRS-R [52]. However, Peters et al. [34] found no association between the ALSFRS-R score and concentrations of plasma copper. Despite the conflicting results, it is known that copper is indirectly involved with motor neuron degeneration mechanisms in ALS by mutation of the copper-dependent enzyme SOD-1, TDP-43 protein aggregation, and mitochondrial dysfunction [39]. Among the serum lipid biomarkers, only TC was inversely associated with the functional status of ALS patients studied. This fact may occur due to possible oxidation of cholesterol mediated by the copper as the disease progresses. On the other hand, the positive association found between the functional status and LDL-c, HDL-c, and TGL corroborates with the possible protective effect of hyperlipidemia in the survival of patients with ALS demonstrated in some studies [8–11].

The main limitations of this study were (1) the small number of participants in both groups due to the disease rarity and difficulty in finding healthy matches and (2) the few numbers of copper biomarkers evaluated. These limitations may have weakened the statistical power of the analysis and restricted deeper inferences in the discussion. Therefore, further studies are needed on this topic, considering larger samples and including other biomarkers of copper status and oxidative stress, as well as the presence of genetic polymorphisms related to lipid metabolism.

## 5. Conclusion

This study suggests a disturbance of copper status and its connection with the lipid profile in patients with ALS. Furthermore, copper status and lipid profile may influence the functional status of patients with ALS, standing out as potential biomarkers of disease severity.

## Figures and Tables

**Table 1:** Clinical, nutritional, and biochemical characteristics of the participants.

Variable	Case group (*n*=27)	Control group (*n*=26)	*p* value
Age (years)^a^	56.0 (12.6)	55.4 (12.5)	0.9989
Sex^c^			
Male	13 (48)	11 (42)	—
Female	14 (52)	15 (58)	—
BMI (kg/m)^2a^	22.6 (2.9)	27.3 (4.7)	<0.0001
Initial manifestation^c^			
Spinal ALS	20 (74)	—	—
Bulbar ALS	7 (26)	—	—
Disease time in months^b^	46 (22–72)	—	—
ALSFRS-R score^b^	21 (14–32)	—	—
>24 points^c^	8 (40)	—	—
≤24 points^c^	12 (60)	—	—
Feeding pathway^c^			
Oral	21 (78)	—	—
Enteral	5 (18)	—	—
Oral + enteral	1 (4)	—	—
Dietary copper (mg/dia)^b^	1.3 (0.7–2.1)	0.9 (0.8–1.1)	0.4122
Plasma copper (*μ*g/dL)^a^	133.9 (26.5)	164.1 (25.7)	0.0001
Serum ceruloplasmin (mg/dL)^a^	23.2 (6.3)	25.0 (4.2)	0.2271
Total cholesterol (mg/dL)^a^	190.6 (45.6)	190.2 (41.9)	0.9761
LDL-cholesterol (mg/dL)^a^	121.3 (38.1)	124.1 (35.9)	0.7877
HDL-cholesterol (mg/dL)^a^	43.7 (9.5)	45.0 (8.6)	0.6162
Triglycerides (mg/dL)^a^	126.0 (55.4)	124.9 (70.9)	0.9527

^a^Mean (standard deviation); ^b^median (interquartile range); ^c^frequency (percentage); BMI: body mass index; ALSFRS-R: ALS Functional Rating Scale-Revised.

**Table 2:** Correlation of plasma copper and serum ceruloplasmin with dietary copper and lipid profile of the participants.

Variable	Case group			Control group		
	*n*	*r*_s_	*p* value	*n*	*r*_s_	*p* value
Plasma copper						
Dietary copper	27	0.194	0.333	23	0.054	0.807
Serum ceruloplasmin	23	0.152	0.488	25	0.646	<0.001
Total cholesterol	27	0.293	0.138	25	0.446	0.025
LDL-cholesterol	27	0.292	0.140	24	0.445	0.029
HDL-cholesterol	26	0.398	0.044	25	0.479	0.015
Triglycerides	27	−0.208	0.297	25	0.371	0.068

Serum ceruloplasmin						
Dietary copper	23	−0.145	0.508	24	−0.184	0.390
Total cholesterol	23	0.083	0.707	26	0.320	0.110
LDL-cholesterol	23	0.053	0.810	25	0.407	0.043
HDL-cholesterol	22	0.211	0.346	26	0.254	0.210
Triglycerides	23	−0.290	0.179	26	0.134	0.515

*r*_s_: Spearman's correlation coefficient.

**Table 3:** Association of Amyotrophic Lateral Sclerosis Functional Rating Scale-Revised (ALSFRS-R) with copper and lipid biomarkers in patients with amyotrophic lateral sclerosis.

Variable	*B*	Standard error	95% CI		Hypothesis testing		
			Inferior	Superior	*χ* ^2^	df	*p* value
Model					79.42	8	<0.001
Interception	3.779	0.4886	2.821	4.736	59.824	1	<0.001
Dietary copper	−0.373	0.0784	−0.527	−0.219	22.646	1	<0.001
Plasma copper	−0.005	0.0023	−0.010	0.000	4.545	1	0.033
Serum ceruloplasmin	0.016	0.0081	0.000	0.032	4.046	1	0.044
Total cholesterol	−0.312	0.0959	−0.500	−0.124	10.611	1	0.001
LDL-cholesterol	0.314	0.0958	0.126	0.502	10.748	1	0.001
HDL-cholesterol	0.308	0.0943	0.123	0.493	10.686	1	0.001
Triglycerides	0.062	0.0190	0.025	0.099	10.568	1	0.001

Dependent variable: ALSFRS-R score. *B*: regression coefficient; 95% CI: 95% confidence interval; *χ*^2^: chi-square test; df: degrees of freedom; *p* value: probability of the null hypothesis is true.

## Data Availability

Data are available upon request.
